# Supplementary material for: Adenoviral delivery of the CIITA transgene induces T‐cell‐mediated killing in glioblastoma organoids
Source: Mol Oncol. 2024 Nov 13;19(3):682–97. doi: 10.1002/1878-0261.13750 (PMC11887676; doi:10.1002/1878-0261.13750)
Supplement: Supplementary file 1 — Fig. S1. MHC‐II related gene expression analysis in GB patient tumors and human preclinical models. Fig. S2. Characterization of wild‐type and mutant CIITA adenoviral vectors in adherent human (U251) and murine (GL261) cell lines. Fig. S3. Infection of human primary glioblastoma organoids with adenoviral vectors and impact on MHC‐I expression. Fig. S4. Evaluation of immune cell‐mediated tumor cell killing in human primary GB organoids. Fig. S5. Requirement for CIITA expression and immune‐tumor cell contact, but not antigen presentation. Fig. S6. Positive controls of neutralization assay. [file MOL2-19-682-s001.zip › FiguresS1-S6_Legends.docx]

**SUPPORTING INFORMATION**

**Supplementary Figure 1. MHC-II related gene expression analysis in GB patient tumors and human preclinical models. A.** Heatmap representing gene expression levels in normal human brain, GB patient tumors, PDOXs, GSC lines (NCH421k, NCH644), and classical glioma lines (U87, U251) grown *in vitro* or *in vivo* as xenograft ('X'). Lack of expression is represented by grey color. Human specific arrays were applied for transcriptome analysis. **B.** Flow cytometric analysis of surface MHC-II (anti-HLA-DR/DP/DQ-FITC) in control conditions (cells unstained or mock-stimulated) and upon IFN-γ stimulation.

**Supplementary Figure 2. Characterization of wild-type and mutant CIITA adenoviral vectors in adherent human (U251) and murine (GL261) cell lines. A.** Sanger sequencing results of the wild-type vs mutant *CIITA* DNA. **B.** PyMOL representation of the amino acid string surrounding position 1088 in wild-type (left) and mutated (right) CIITA. **C.** Representative confocal images of CIITA (Alexa Fluor 488) in U251 at 72 hours post-infection (magnification 63X). CIITA cellular distribution was quantified by measuring the ratio of cytoplasmic-to-nuclear fluorescence intensity. n=6. Mean ± SD. ****p<0.0001 (two-way ANOVA). **D-F.** Ad-CIITA characterization in GL261. **D.** Flow cytometric analysis of MHC-II (anti-IA-IE-PerCP) at increasing Ad-CIITA MOIs. n=3. **E.** mRNA (left, n=3) and protein (right, n=1) levels of CIITA at 48 and 72 hours post-infection, as analyzed by qRT‒PCR and Western blot, respectively. qRT-PCR and WB results were normalized to GAPDH. Mean ± SD. ***p<0.001, ****p<0.0001 (two-way ANOVA).

**Supplementary Figure 3. Infection of human primary glioblastoma organoids with adenoviral vectors and impact on MHC-I expression. A.** Flow cytometric analysis of surface MHC-I (anti-HLA-A/B/C-BV510), CD80 (anti-CD80-PE) and CD86 (anti-CD86-BV421) in P3 organoids. **B.** Tumor cell viability of T16dsRed (top) and red dye-labeled T188 (bottom) organoids infected at increasing virus MOIs. n=3. Mean ± SEM.

**Supplementary Figure 4. Evaluation of immune cell-mediated tumor cell killing in human primary GB organoids. A.** Flow cytometry gating strategy to phenotype PBMCs and MACS-purified T-cell fractions. **B-C.** Tumor cell viability in T16dsRed (left panel, MOI 25) or red dye-labeled T188 (right panel, MOI 75) organoids: **(B)** alone (no co-culture) or co-cultured with PBMCs; **(C)** co-cultured with CD3^+^, CD4^+^, or CD8^+^ T-cells. **D.** Tumor cell viability in T16dsRed organoids (MOI 25) co-cultured with HLA-matched PBMCs or CD3^+^ T-cells. n=3. Mean ± SEM. *p<0.05, **p<0.01, ***p<0.001, and ****p<0.0001 (two-way ANOVA). Deviations from virus MOI are explicitly indicated on the graph.

**Supplementary Figure 5. Requirement for CIITA expression and immune-tumor cell contact, but not antigen presentation. A-C.** T16dsRed organoids (MOI 25) co-cultured with PBMCs (T:E - 1:10). **A.** GFP expression in Ad-GFP-infected vs non-infected organoids. n=3. Mean ± SD. ****p<0.0001 (two-way ANOVA). **B.** Tumor cell viability in Ad-GFP- or Ad-CIITA-infected GB organoids compared to non-infected control. n=3. Mean ± SEM. *p<0.05, **p<0.01 (one-way ANOVA). **C.** Tumor cell viability in two-layer transwell co-culture model. n=3. Mean ± SEM.

**Supplementary Figure 6. Positive controls of neutralization assay.** Actinomycin D- and DMSO-induced tumor cell death blocked by anti-human TNF-R (MAB225) (left panel) and anti-human FasL (MAB126) (right panel), respectively. n=3. Mean ± SEM.

**Supplementary Table 1. Tumor/Immune cells typing at MHC-I and MHC-II loci.**

**Supplementary Table 2. Composition of PBMC and isolated sub-populations before co-culture (shown as % of CD45⁺).**

**Supplementary Table 3. List of all antibodies used in the study.**

**Supplementary Table 4. Molecular profiling of the PDOX models used in the study.**

**Video S1. Organoid disruption and tumor cell killing in Ad-CIITA-infected and Ad-CIITA mutant-infected primary GB organoids co-cultured with PBMCs.**
