# Supplementary figures and images for: Adenoviral delivery of the CIITA transgene induces T‐cell‐mediated killing in glioblastoma organoids
Source: Mol Oncol. 2024 Nov 13;19(3):682–97. doi: 10.1002/1878-0261.13750 (PMC11887676; doi:10.1002/1878-0261.13750)

# B

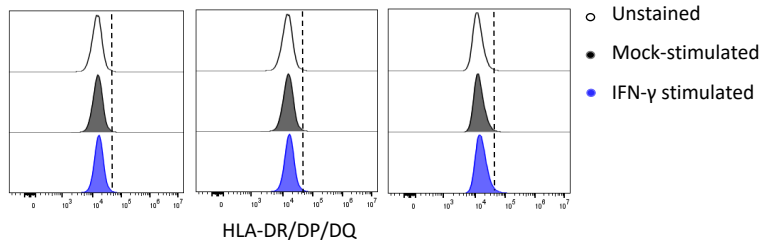

Supplement: Supplementary file 1 — Fig. S1. MHC‐II related gene expression analysis in GB patient tumors and human preclinical models. Fig. S2. Characterization of wild‐type and mutant CIITA adenoviral vectors in adherent human (U251) and murine (GL261) cell lines. Fig. S3. Infection of human primary glioblastoma organoids with adenoviral vectors and impact on MHC‐I expression. Fig. S4. Evaluation of immune cell‐mediated tumor cell killing in human primary GB organoids. Fig. S5. Requirement for CIITA expression and immune‐tumor cell contact, but not antigen presentation. Fig. S6. Positive controls of neutralization assay. [file MOL2-19-682-s001.zip › FigureS1.pdf]

**A**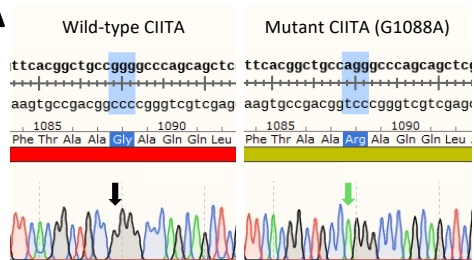**B**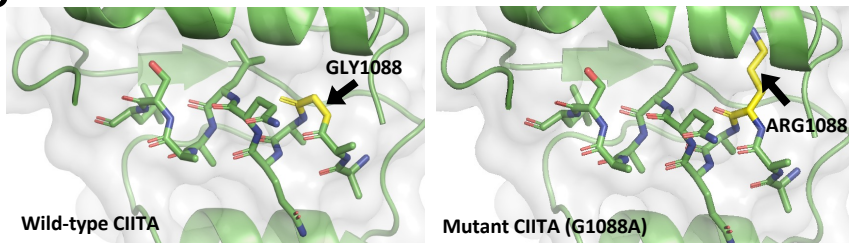**C**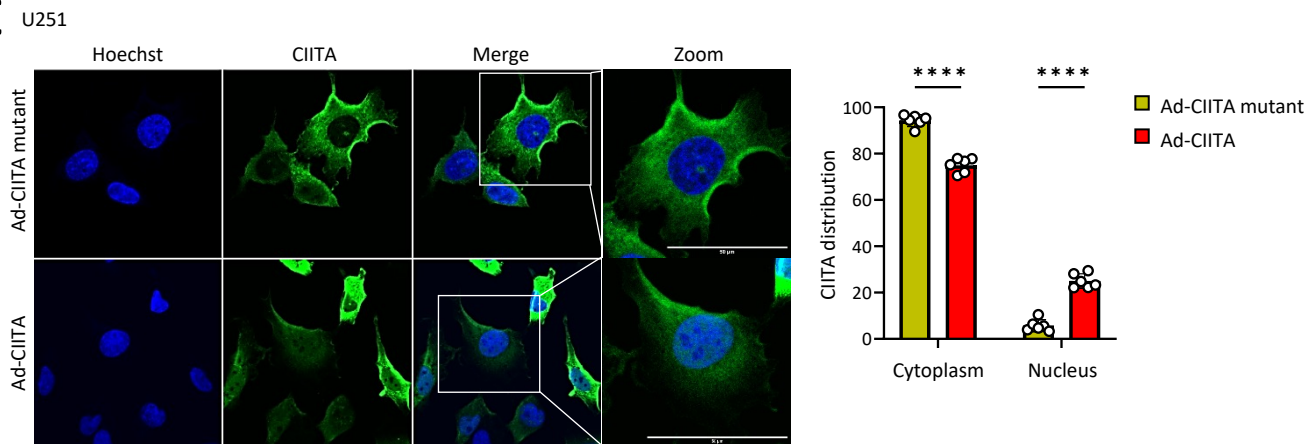**D**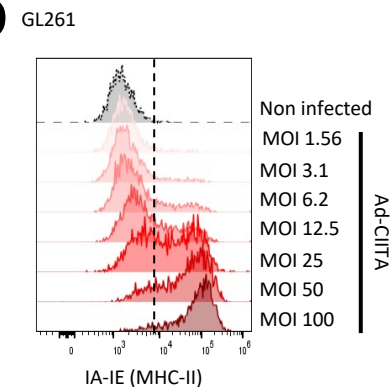**E**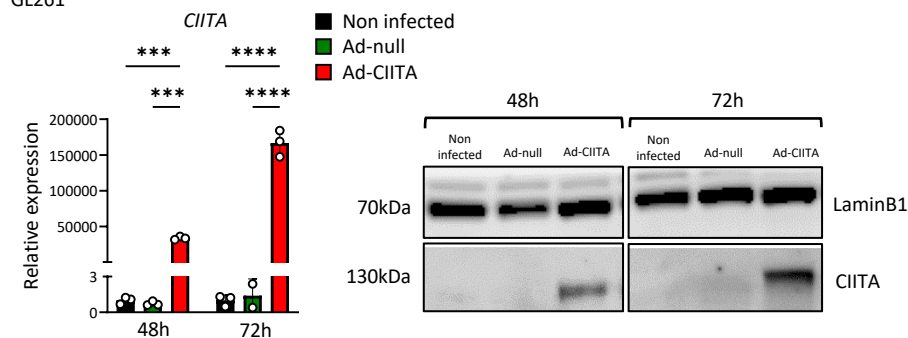

Supplement: Supplementary file 1 — Fig. S1. MHC‐II related gene expression analysis in GB patient tumors and human preclinical models. Fig. S2. Characterization of wild‐type and mutant CIITA adenoviral vectors in adherent human (U251) and murine (GL261) cell lines. Fig. S3. Infection of human primary glioblastoma organoids with adenoviral vectors and impact on MHC‐I expression. Fig. S4. Evaluation of immune cell‐mediated tumor cell killing in human primary GB organoids. Fig. S5. Requirement for CIITA expression and immune‐tumor cell contact, but not antigen presentation. Fig. S6. Positive controls of neutralization assay. [file MOL2-19-682-s001.zip › FigureS2.pdf]

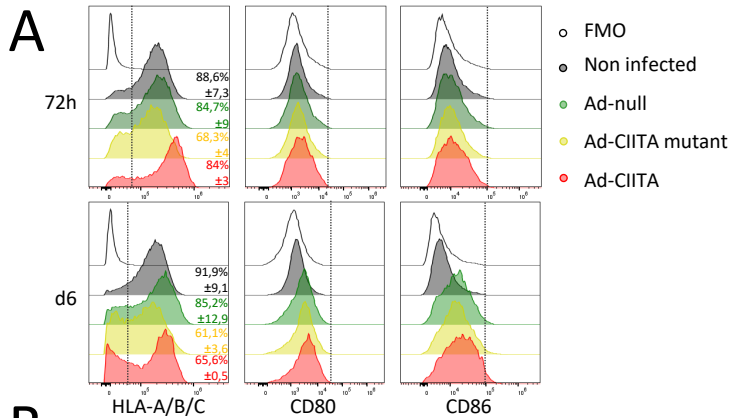

**B**

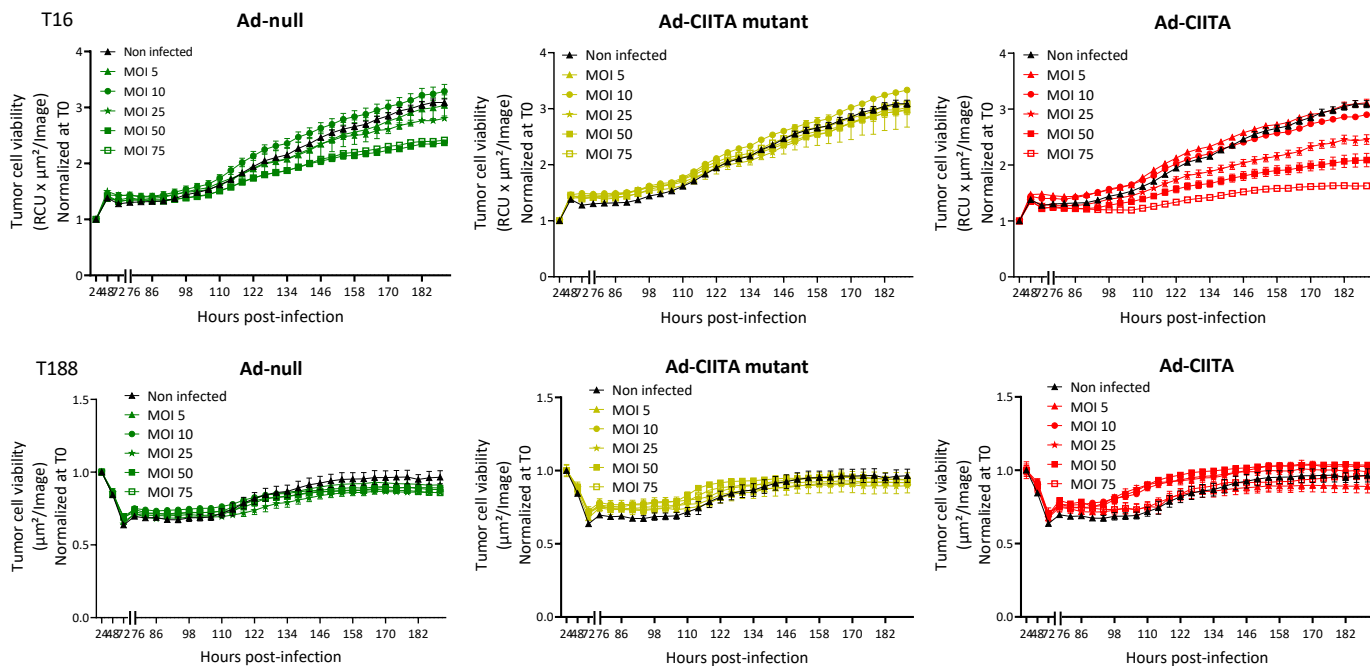

Supplement: Supplementary file 1 — Fig. S1. MHC‐II related gene expression analysis in GB patient tumors and human preclinical models. Fig. S2. Characterization of wild‐type and mutant CIITA adenoviral vectors in adherent human (U251) and murine (GL261) cell lines. Fig. S3. Infection of human primary glioblastoma organoids with adenoviral vectors and impact on MHC‐I expression. Fig. S4. Evaluation of immune cell‐mediated tumor cell killing in human primary GB organoids. Fig. S5. Requirement for CIITA expression and immune‐tumor cell contact, but not antigen presentation. Fig. S6. Positive controls of neutralization assay. [file MOL2-19-682-s001.zip › FigureS3.pdf]

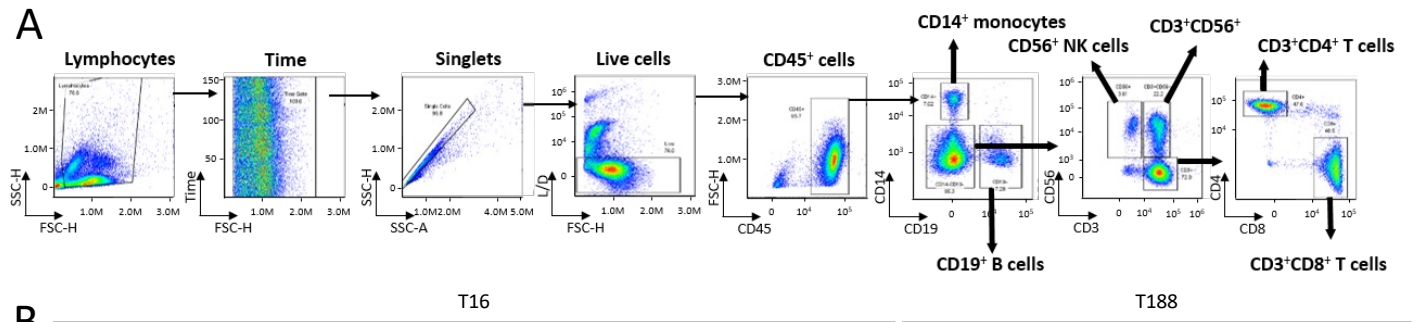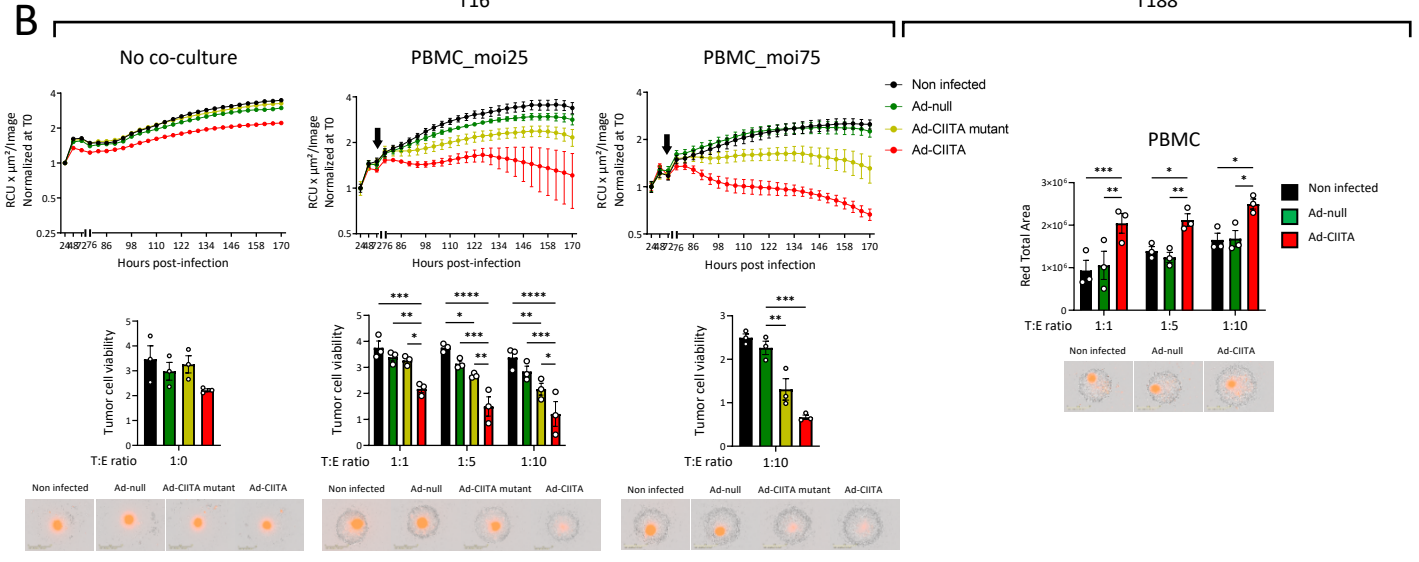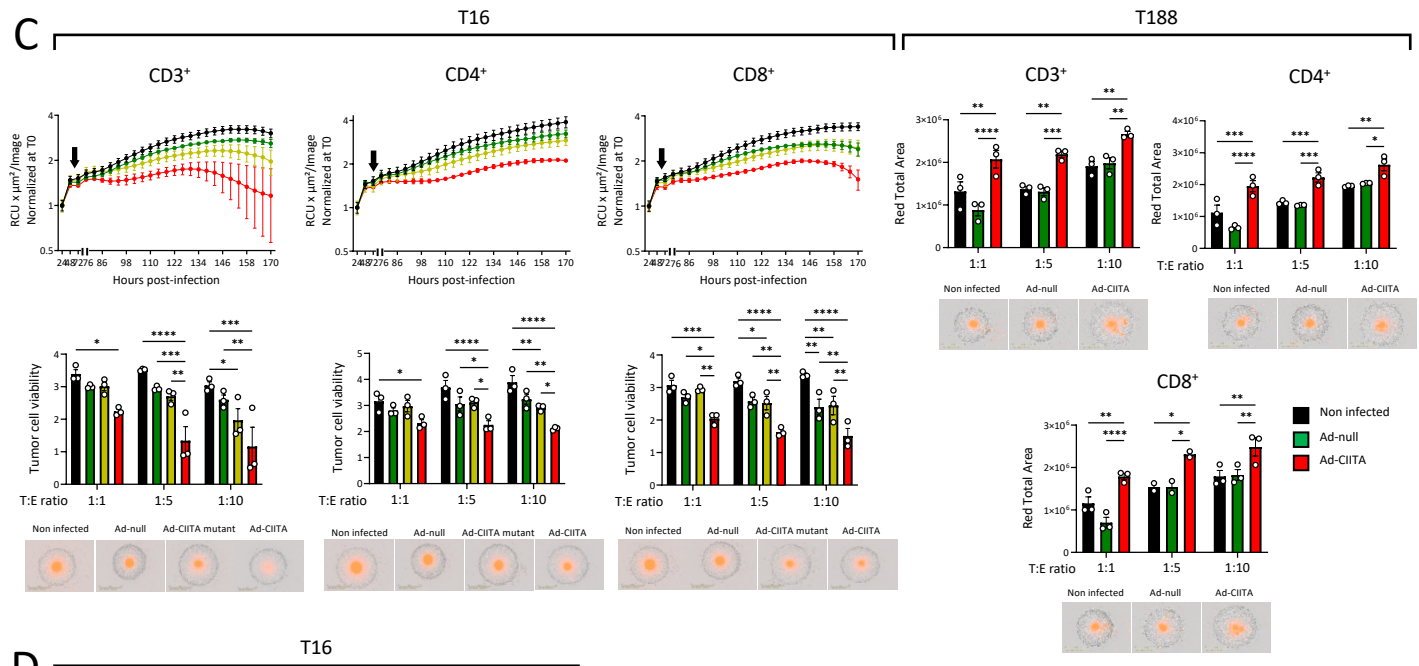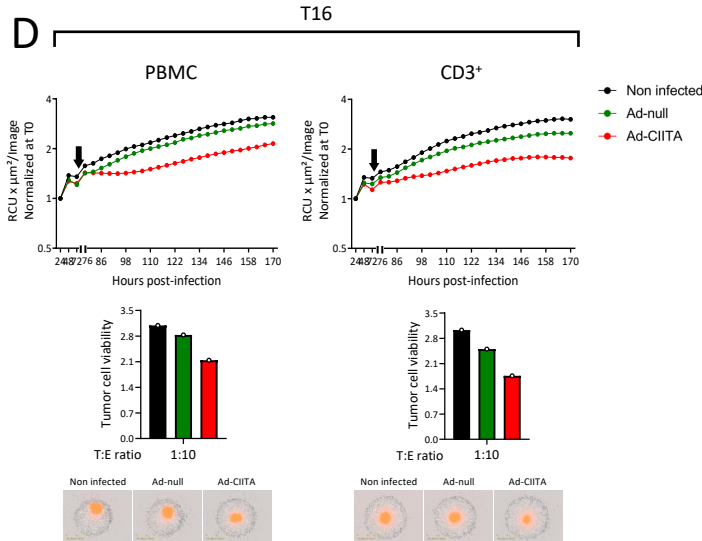

Supplement: Supplementary file 1 — Fig. S1. MHC‐II related gene expression analysis in GB patient tumors and human preclinical models. Fig. S2. Characterization of wild‐type and mutant CIITA adenoviral vectors in adherent human (U251) and murine (GL261) cell lines. Fig. S3. Infection of human primary glioblastoma organoids with adenoviral vectors and impact on MHC‐I expression. Fig. S4. Evaluation of immune cell‐mediated tumor cell killing in human primary GB organoids. Fig. S5. Requirement for CIITA expression and immune‐tumor cell contact, but not antigen presentation. Fig. S6. Positive controls of neutralization assay. [file MOL2-19-682-s001.zip › FigureS4.pdf]

**A**

T16

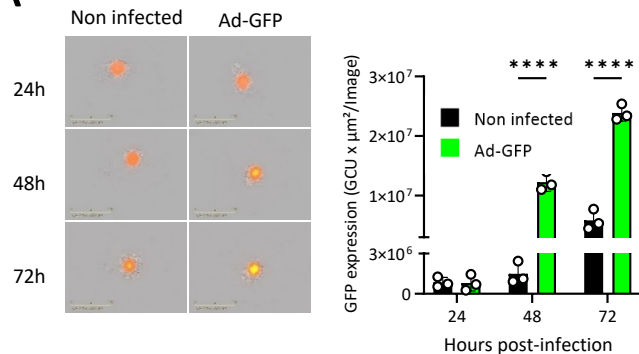**B**

T16

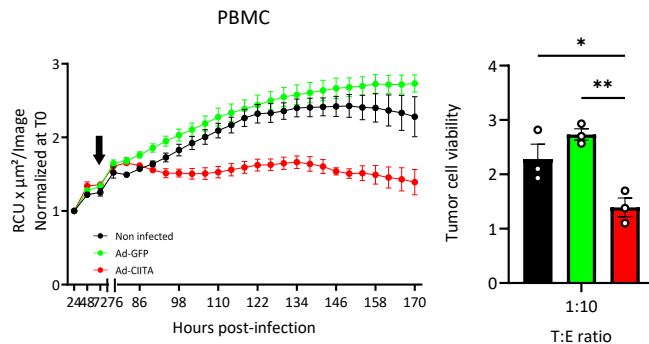**C**

T16

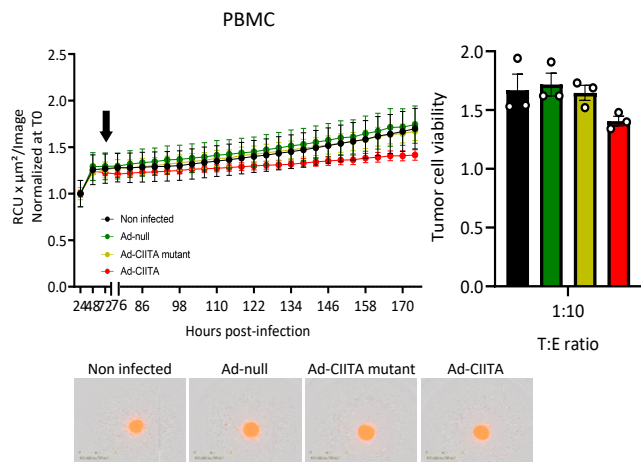

Supplement: Supplementary file 1 — Fig. S1. MHC‐II related gene expression analysis in GB patient tumors and human preclinical models. Fig. S2. Characterization of wild‐type and mutant CIITA adenoviral vectors in adherent human (U251) and murine (GL261) cell lines. Fig. S3. Infection of human primary glioblastoma organoids with adenoviral vectors and impact on MHC‐I expression. Fig. S4. Evaluation of immune cell‐mediated tumor cell killing in human primary GB organoids. Fig. S5. Requirement for CIITA expression and immune‐tumor cell contact, but not antigen presentation. Fig. S6. Positive controls of neutralization assay. [file MOL2-19-682-s001.zip › FigureS5.pdf]

P3

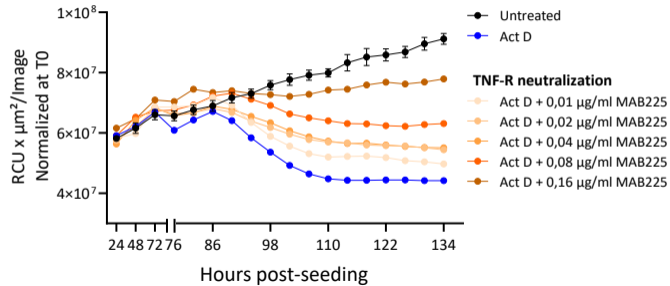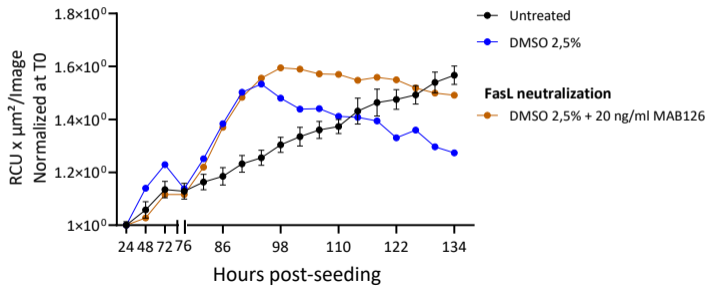

Supplement: Supplementary file 1 — Fig. S1. MHC‐II related gene expression analysis in GB patient tumors and human preclinical models. Fig. S2. Characterization of wild‐type and mutant CIITA adenoviral vectors in adherent human (U251) and murine (GL261) cell lines. Fig. S3. Infection of human primary glioblastoma organoids with adenoviral vectors and impact on MHC‐I expression. Fig. S4. Evaluation of immune cell‐mediated tumor cell killing in human primary GB organoids. Fig. S5. Requirement for CIITA expression and immune‐tumor cell contact, but not antigen presentation. Fig. S6. Positive controls of neutralization assay. [file MOL2-19-682-s001.zip › FigureS6.pdf]
